# Immunogenicity of recombinant vesicular stomatitis virus-vectored vaccines expressing the Machupo virus glycoprotein in different genome positions

**DOI:** 10.1128/jvi.00357-26

**Published:** 2026-07-24

**Authors:** Rachel Erickson, Paul Bates

**Affiliations:** 1 Department of Microbiology, Perelman School of Medicine, University of Pennsylvania 6572, Philadelphia, Pennsylvania, USA; University of Kentucky College of Medicine, Lexington, Kentucky, USA

**Keywords:** Machupo virus, recombinant VSV vaccines, new world arenavirus, Junín virus, VSV genome rearrangements, humoral response, cellular response

## Abstract

Recombinant vesicular stomatitis virus (rVSV) is a powerful vaccine platform that elicits durable cellular and humoral immune responses. rVSV vaccines have successfully been used for various hemorrhagic fever-causing viruses. Here, we create and evaluate rVSV vaccines for Machupo virus (MACV), a highly pathogenic New World (NW) mammarenavirus that causes Bolivian hemorrhagic fever. VSV genome rearrangements impact attenuation of the viral vector and the immune response to the vaccine. Therefore, in this study, we directly compare the effect of altering the MACV glycoprotein complex (GPC) position in the VSV genome on humoral and cellular immune responses to vaccination by evaluating replication, antigen expression, and immunogenicity of rVSVs expressing MACV GPC in the first position of the genome (rVSV-MACV1) and in the traditional glycoprotein gene position (rVSV-MACV5). rVSV-MACV1 expresses higher levels of MACV GPC in infected cells and in virions, but the increased antigen does not translate to a more robust immune response. rVSV-MACV5 elicits higher levels of total IgG and neutralizing antibodies than rVSV-MACV1, but similar levels of MACV-specific T cells. To further investigate vaccine strategies for MACV and other NW arenaviruses such as Junín virus (JUNV), we evaluated the humoral immune response to sequential immunization with rVSVs expressing MACV and JUNV GPCs. The highest levels of MACV and JUNV reactive antibodies were achieved using an rVSV-MACV5 prime followed by rVSV-JUNV boost. The data from our study have implications for rVSV vaccine design and provide a foundation for the development of rVSV-vectored vaccines for MACV and other NW arenaviruses.

**IMPORTANCE:**

Machupo virus (MACV) is a highly pathogenic New World (NW) mammarenavirus that causes Bolivian hemorrhagic fever (BHF). Because of their high mortality rates, MACV and other NW arenaviruses represent a significant public health threat. However, no approved vaccines for MACV exist. Here, we describe recombinant vesicular stomatitis virus (rVSV) vaccines that express the MACV glycoprotein complex (GPC) in different positions of the VSV genome and characterize the humoral and cellular immune response to each. We also investigate sequential immunization with rVSVs expressing MACV GPC and a related NW arenaviral antigen, Junín virus GPC, in an effort to elicit broadly cross-reactive antibody responses. The conclusions from our study are twofold: data presented here provide further understanding of rVSV vaccine vectors and how genome rearrangements can be leveraged for vaccine development, and the results from our study may inform future vaccine development for MACV and other NW arenaviruses.

## INTRODUCTION

Machupo virus (MACV), a highly pathogenic New World (NW) arenavirus, is the causative agent of Bolivian hemorrhagic fever (BHF). BHF begins with a non-specific flu-like illness that can progress to late-stage hemorrhagic fever. The overall mortality rate of BHF is approximately 30%, and there are currently no FDA-approved vaccines or therapeutics. Its virulence and potential for aerosol transmission make MACV a public health and biodefense concern. As a result, the NIH has classified MACV as a Category A priority pathogen, a designation that highlights the critical need for vaccine development (1).

MACV is one of several hemorrhagic fever-causing mammarenaviruses. Members of the genus *Mammarenavirus* of the *Arenaviridae* family are classified into New World and Old World (OW) arenaviruses according to geographical location and phylogenetic relationships (2). Argentine hemorrhagic fever (AHF) caused by Junín virus (JUNV) and BHF caused by MACV are the most common NW arenavirus infections in South America (3). Lassa fever (LF), caused by Lassa virus (LASV), is the most prevalent OW arenavirus infection in Africa, causing between 100,000 and 300,000 infections annually (4). Despite their threat to public health, there is only one vaccine for arenaviruses, a live attenuated vaccine for JUNV called Candid#1 (2). However, there are significant concerns about reversion to virulence, and this vaccine is only approved for limited populations in Argentina, where JUNV is endemic (5).

Few MACV-specific vaccines have been developed. Most vaccines focus on developing an immune response to the MACV glycoprotein complex (GPC), which is the only viral surface protein and is thought to be the target of protective immunity. The MACV glycoprotein precursor is cleaved into three parts: the stable signal peptide, which remains associated with the GP, forming a heterotrimer; GP1, which is the receptor-binding domain; and GP2, which mediates viral fusion (6). Tested vaccine platforms include live attenuated, recombinant virus (7), and alphavirus RNA replicon vector vaccines (8). Due to the structural similarities and genetic relatedness of MACV and JUNV, attenuated MACV vaccines have been created by introducing mutations in MACV GPC that correspond to those in the GPC of JUNV Candid#1 (9). Additionally, a recombinant MACV expressing the JUNV Candid#1 GPC was shown to be attenuated and protective in IFN-αβ/γ R^−/−^ mice (7). Another recent vaccine used a highly attenuated Mopeia arenavirus as a viral vector to express either the MACV GPC or five different arenaviral GPCs, including MACV GPC; both constructs were safe and protected guinea pigs from lethal MACV challenge (10). Despite their success in small animal models, none of these vaccines has been evaluated in humans.

Replication-competent viral vectored vaccines are prized for their ability to induce long-lasting immune responses. Vesicular stomatitis virus (VSV) is a single-stranded negative-sense RNA virus. Recombinant VSVs (rVSVs) expressing and incorporating foreign glycoproteins have been developed as vaccines for several diseases, including hemorrhagic fevers (11). For example, a vaccine for Ebola virus (EBOV) disease was created by replacing the VSV glycoprotein (G) with the EBOV glycoprotein (GP) (12). This vaccine was FDA-approved in 2019 and has been successfully administered to over 350,000 people (13). A single dose of the rVSV-EBOV vaccine had an efficacy rate of 100% in clinical trials (14) and 84% in the 2018–2019 outbreak in the Democratic Republic of the Congo (15). Furthermore, VSV vaccines have been shown to provide rapid protection against lethal challenge, with the rVSV-EBOV vaccine providing partial protection within 3 days and complete protection within 7 days in an NHP study (16). A similar rVSV vector that expresses the LASV glycoprotein instead of EBOV GP in place of VSV G has been used to develop a vaccine for LF; rVSV-LASV has been shown to be safe and 100% effective in protecting non-human primates (NHPs) from lethal LASV challenge (17). rVSV-LASV was shown to be safe and immunogenic in early clinical trials (18) and is currently in phase II clinical trials in West Africa (19). The success of rVSV-LASV suggests that the rVSV platform may be useful in creating vaccines for other arenaviruses such as MACV. Additionally, studies in NHPs have shown that prior immunization with rVSV-LASV does not interfere with mounting a protective immune response to rVSV-EBOV, indicating that sequential immunization with multiple rVSV vaccines is possible (20). As replication-competent viruses, rVSV vectored vaccines induce strong type 1 immune responses, which are ideal for the control and elimination of intracellular pathogens such as viruses (21–24). Moreover, immune responses to rVSV vaccines in humans are extremely durable. Studies have reported long-lived B- and T-cell responses up to 5 years after vaccination with rVSV-EBOV (25, 26).

In addition to expressing foreign genes in place of VSV G, the VSV genome can also tolerate gene order rearrangements and insertions of additional open reading frames (ORFs) that attenuate the virus and modulate gene expression (27–30). The VSV genome encodes five viral proteins (in order from 3′ to 5′): nucleoprotein (N), phosphoprotein (P), matrix protein (M), glycoprotein (G), and RNA-dependent RNA polymerase (L) (31, 32). VSV genes are transcribed in a 3′ to 5′ gradient with a 30% decrease in transcript number for each subsequent gene (33). It has been shown that expression of the VSV G protein in the first position of the genome resulted in an increased amount of G-infected cells and virions, as well as an accelerated and enhanced anti-G humoral immune response in mice (34). We hypothesized that we could leverage gene order rearrangements to produce rVSV vaccines that had these desirable characteristics for vaccine candidates, such as a more rapid and robust humoral immune response.

Here, we present data on the recovery and characterization of an rVSV expressing the MACV GPC in the first position of the genome (rVSV-MACV1) and compare it to an rVSV expressing MACV GPC in the traditional gene position (rVSV-MACV5). We hypothesized that rVSV-MACV1 would elicit higher levels of antibodies due to increased antigen delivery. We evaluated the growth of the rVSVs *in vitro* and *in vivo* and examined the immunogenicity of these vaccine candidates in C57BL/6 mice by characterizing the humoral and cellular immune response to vaccination. We found that despite the increased antigen delivery by rVSV-MACV1, it did not translate to a more robust immune response. rVSV-MACV5 induced higher levels of total IgG and neutralizing antibodies in mice, and there was no significant difference in cellular immune response to the two vaccine constructs. Additionally, we evaluated the breadth of the humoral immune response to rVSV-MACV5 compared to and in combination with rVSV-JUNV. Together, these data suggest that rVSV is a promising platform for the development of a MACV vaccine.

## RESULTS

### Generation and characterization of rVSV-MACV vaccines

**Fig 1:**
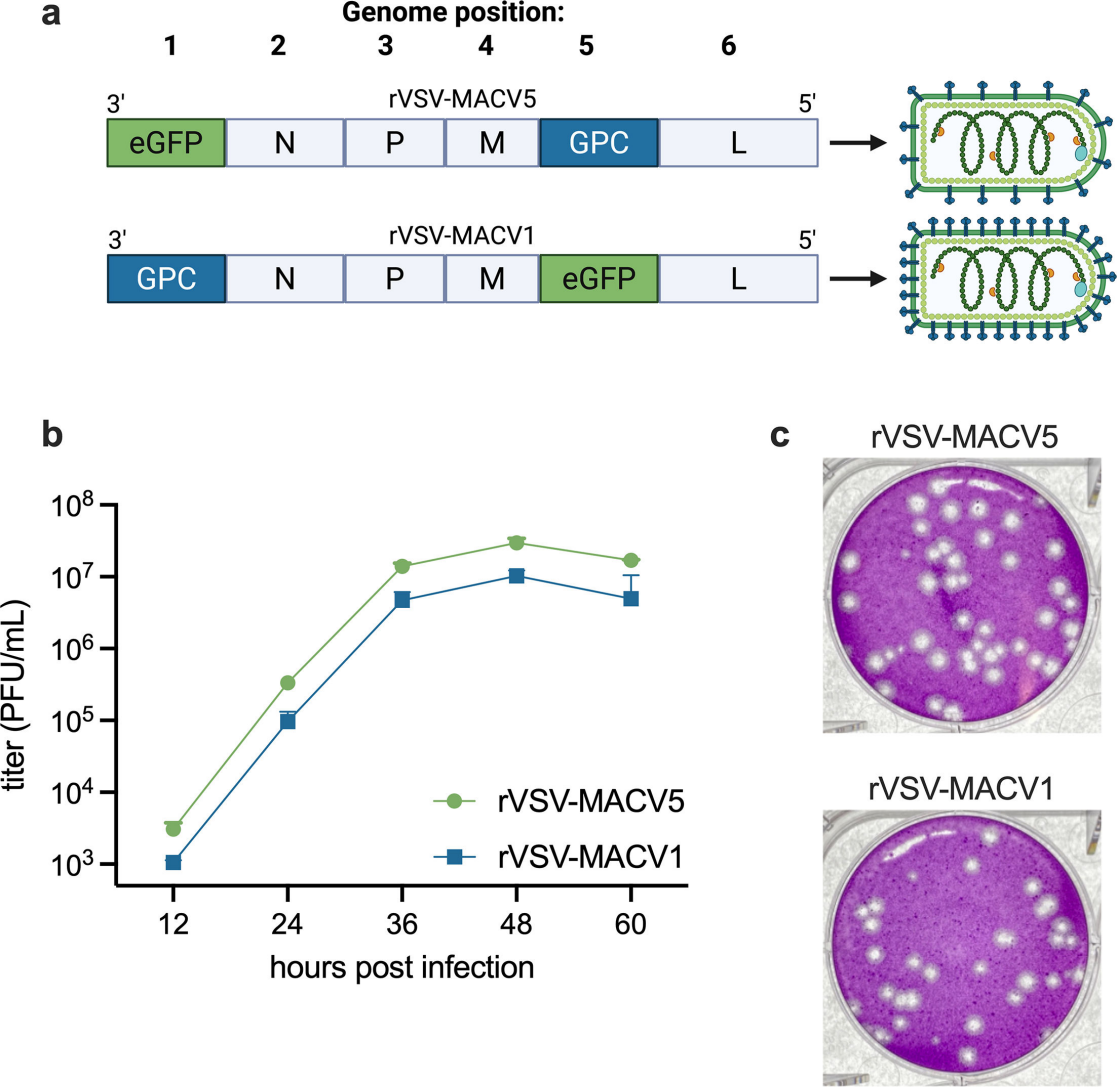
rVSV-MACV5 and rVSV-MACV1 vaccine design and characterization. (**a**) Schematic of the recombinant VSV genomes containing eGFP, VSV nucleoprotein (N), VSV phosphoprotein (P), VSV matrix protein (M), MACV GPC, VSV polymerase (L), and illustration of rVSV-MACV virions expressing MACV GPC on the surface. rVSV-MACV5 expresses MACV GPC in genome position 5; rVSV-MACV1 expresses MACV GPC in genome position 1. Created using BioRender.com. (**b**) Growth kinetics of rVSV-MACV5 (green circles) and rVSV-MACV1 (blue squares) in Vero E6 cells infected at a multiplicity of infection (MOI) of 0.0001. Supernatants were collected every 12 hours, and viral titer was evaluated via plaque assay. Data show mean ± standard deviation from two independent experiments. (**c**) Representative images of plaques formed by rVSV-MACV5 (top) and rVSV-MACV1 (bottom) on Vero E6 cells.

The rVSV genomes were generated using restriction cloning to insert a codon-optimized MACV GPC sequence in the fifth and first genome positions of the VSV genome. In other published examples of VSV genome rearrangements, placing the GP in position 1 inevitably shifted N to position 2 and resulted in attenuation of viral replication. To create a system where the effect of altering GP placement within the genome could be directly compared, we included an additional ORF encoding eGFP. The resultant viruses express N, P, M, and L in positions 2, 3, 4, and 6, respectively, in both viral vectors with eGFP and MACV GPC in either position 1 or 2 (Fig. 1a). The vectors were named rVSV-MACV5 and rVSV-MACV1 based on the position from which MACV GPC is expressed. We successfully rescued replication-competent rVSV-MACV5 and rVSV-MACV1 using a previously described reverse genetics system (35).

To evaluate replication of the viruses, we infected Vero E6 cells at an MOI of 0.0001 and collected samples at 12-hour intervals post-infection. Infectious titer was quantified by plaque assay on Vero E6 cells. Both viruses replicated well, producing titers between 10^6^ and 10^7^ PFU/mL. Replication kinetics of the two viruses were similar, although rVSV-MACV5 consistently reached titers that were about half a log higher than rVSV-MACV1. Both rVSV-MACV5 and rVSV-MACV1 reached peak titer at 48 hours post-infection (Fig. 1b). Additionally, the plaques formed in a Vero E6 monolayer were of similar size and morphology (Fig. 1c). To evaluate the stability of the MACV GPC in the VSV vector, rVSV-MACV5 was passaged six times on Vero E6 cells and was sequenced after each passage. No mutations were observed in the sequence of MACV GPC after six passages. Overall, we conclude that placement of MACV GPC in either the first or fifth genomic position generates a vector that is stable and replicates well.

### MACV GPC expression in rVSVs

**Fig 2:**
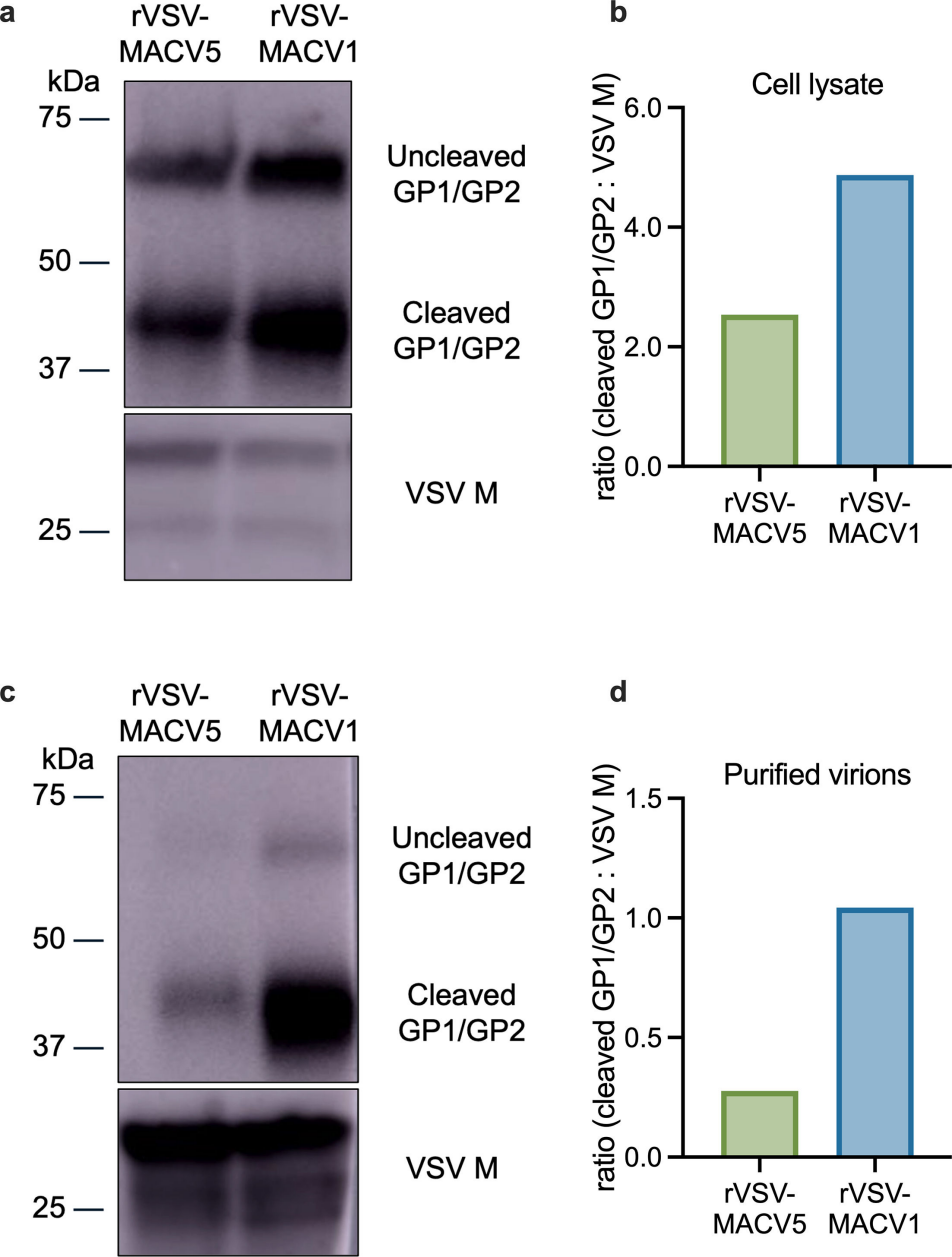
Protein expression in rVSV-MACV5 and rVSV-MACV1. Western blot showing expression of MACV GPC (including uncleaved GP1/GP2 [top] and cleaved GP1/GP2 [bottom]) and VSV M proteins in (**a**) infected Vero E6 cell lysates and (**c**) purified virions. Quantification of cleaved MACV GP1/GP2 relative to VSV M in (**b**) infected Vero E6 cell lysates and (**d**) purified viral particles. ImageJ was used to quantify the intensity of bands on Western blot images.

Based on prior reports (34), we anticipated that there would be a significant difference in MACV GPC expression in rVSV-MACV5 and rVSV-MACV1-infected cells as a result of expressing MACV GPC in different genome positions. To compare MACV GPC protein expression, Vero E6 cells were infected at an MOI of 0.0001 with rVSV-MACV1 and rVSV-MACV5, and media and cell lysates were harvested at 48 hours post-infection. Protein expression was quantified by Western blot analysis of infected cell lysates for both MACV GPC and VSV M proteins. Western blotting showed two bands for MACV GPC: uncleaved GP1/GP2 at ~65 kDa and the cleaved GP1 and GP2, both at ~40 kDa (Fig. 2a). The bands matched previously reported SDS-PAGE data regarding the molecular mass of uncleaved GP1/GP2 and cleaved GP1 and GP2 (36). The ratio of cleaved MACV GP1/GP2 to VSV M protein is approximately 2- to 3-fold higher for rVSV-MACV1 in cell lysates, indicating higher expression levels of MACV GPC in cells infected with rVSV-MACV1 compared to rVSV-MACV5 (Fig. 2b).

To determine whether higher levels of cellular MACV GPC expression affected incorporation of glycoproteins into budded virions, we similarly analyzed protein levels in purified viral particles from the supernatant of infected cells. Protein expression for MACV GP1/GP2 and VSV M in gradient-purified virions was quantified using Western blot analysis. As expected, budded viral particles have a higher ratio of cleaved to uncleaved MACV GP1/GP2 than infected cells (Fig. 2c). Additionally, the ratio of cleaved GP1/GP2 to VSV M was higher for rVSV-MACV1 particles than for rVSV-MACV5 (Fig. 2d). These data are consistent with the transcriptional gradient exhibited by VSV, with higher expression of MACV GPC in rVSV-MACV1 as it is expressed in the first genome position.

### Humoral immune response to rVSV-MACV vaccines in mice

**Fig 3:**
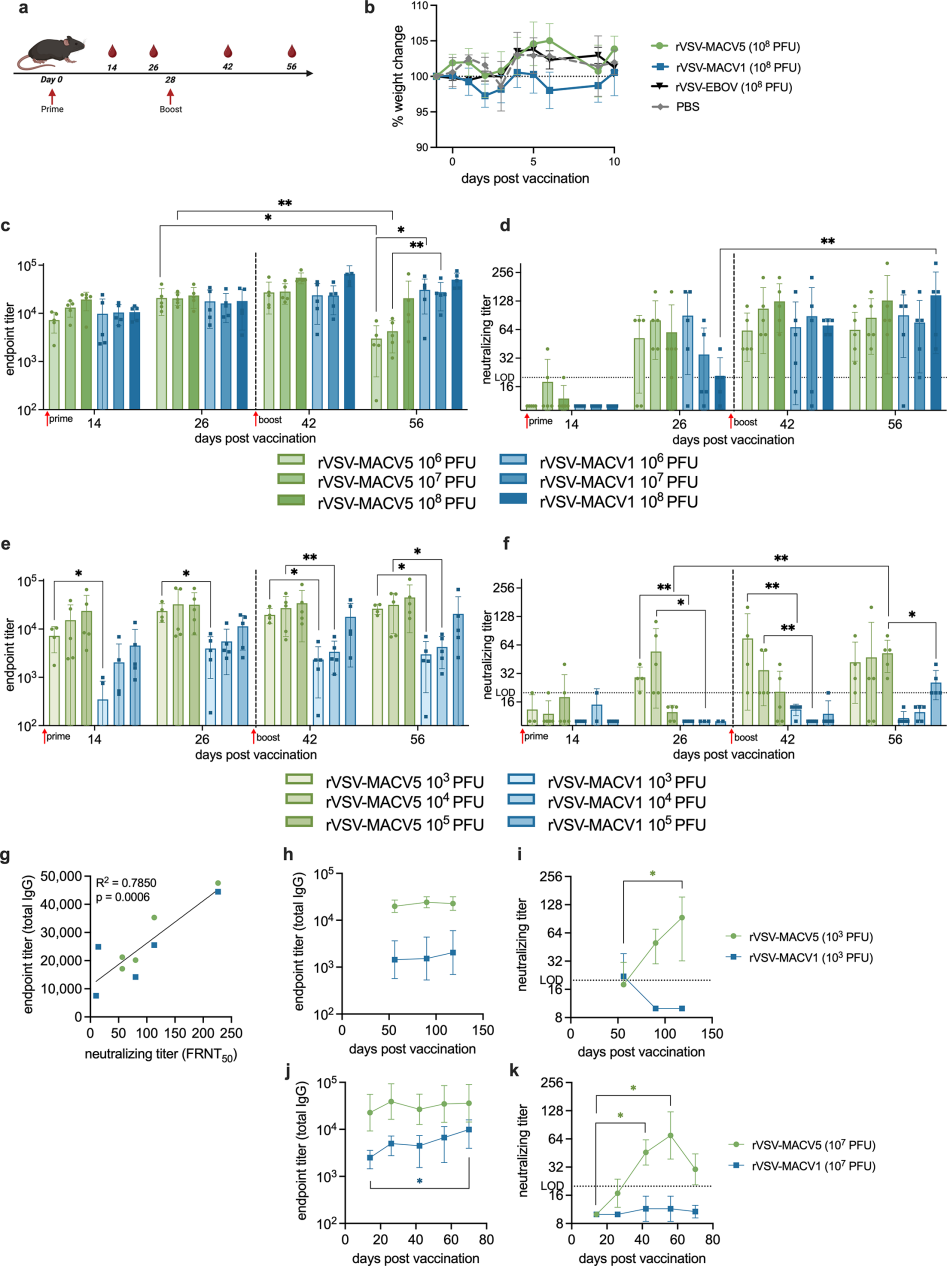
Immunogenicity of rVSV-MACV5 and rVSV-MACV1 in mice. (**a**) Timeline for rVSV-MACV vaccination in C57BL/6 mice (*n* = 5 per group). Mice were vaccinated with either rVSV-MACV5 or rVSV-MACV1 on day 0 and then boosted 28 days later. To allow for VSV replication, on days −1 and +1 relative to vaccination, an anti-interferon-alpha/beta receptor (anti-IFNAR) blocking antibody (0.5 μg/mouse) was administered. The timing of blood collection is indicated by red droplets, which took place every 2 weeks for serological analysis of antibody titers. Panel a was created using BioRender.com. (**b**) Weight change in C57BL/6 mice immunized with 10^8^ PFU of rVSV-MACV5 (green), rVSV-MACV1 (blue), rVSV-EBOV (black), or mock immunized with PBS (gray dotted line). (**c**) Total MACV-specific IgG titers and (**d**) neutralizing titers in serum from mice vaccinated with 10^6^, 10^7^, or 10^8^ PFU of rVSV-MACV5 (green circles) or rVSV-MACV1 (blue squares). (**e**) Total MACV-specific IgG titers and (**f**) neutralizing titers in serum from mice vaccinated with 10^3^, 10^4^, or 10^5^ PFU of rVSV-MACV5 or rVSV-MACV1. Red arrows along the X-axis indicate the timing of prime and boost immunizations. The height of the bar indicates the geometric mean of the group, and individual animals are represented by individual symbols. Each point represents the mean (for endpoint titer/ELISA) or geometric mean (for neutralizing titer/FRNT) from 2 to 3 independent ELISA or FRNT analyses of each serum sample. Statistical analyses were performed to compare titers between different vaccination groups on individual days. Analyses were also performed to compare titers approximately 4 weeks post-immunization; day 26 and day 56, respectively, for prime or prime-boost. When data met the assumption of normality, Welch’s t test was performed; otherwise, the Mann–Whitney test was used, *P < 0.05, **P < 0.01. Lack of illustrated comparison bar between comparison groups indicates no significance between groups, where *P* > 0.05. LOD indicates the limit of detection of the assay. (**g**) Day 42 IgG vs. FRNT_50_ titers from mice receiving 10^7^ PFU prime-boost immunizations. Each symbol represents an individual animal. Simple linear regression, *R*^2^ = 0.7850, *P* = 0.0006. Durability of total IgG (**h**) and neutralizing antibody response (**i**) in serum from mice immunized with two doses of rVSV-MACV5 or rVSV-MACV1 (10^3^ PFU). Durability of total IgG (**j**) and neutralizing antibody response (**k**) in serum from mice immunized with a single dose of rVSV-MACV5 or rVSV-MACV1 (10^7^ PFU). Data show mean ± standard deviation of the group, where titers for each animal were determined from 2 to 3 independent ELISA or FRNT analyses of each serum sample. Statistical analyses were performed to compare antibody titers in each vaccine group over time. When data met the assumption of normality, one-way ANOVA with Tukey’s multiple comparisons test was performed; otherwise, the Kruskal–Wallis with Dunn’s multiple comparisons test was used, *P < 0.05, **P < 0.01. Lack of an illustrated comparison bar between comparison groups indicates no significance between groups, where *P* > 0.05.

We hypothesized that the increased MACV GPC antigen in rVSV-MACV1 would elicit a more robust antibody response in rVSV-immunized mice. To evaluate the immunogenicity of the rVSV-MACV vaccines, we immunized C57BL/6 mice. Groups of five mice each were immunized intraperitoneally (i.p.) with 10^6^, 10^7^, and 10^8^ PFU of rVSV-MACV5 or rVSV-MACV1 on day 0 of the study. Groups of mice were also given 10^8^ PFU of rVSV-EBOV or PBS as controls. All mice received a second dose (boost), administered on day 28, that was identical to the initial vaccination. VSV replication is strongly affected by murine interferon responses (37). Therefore, to allow efficient VSV replication, 0.5 μg of an anti-interferon-alpha/beta receptor (anti-IFNAR) blocking antibody was administered 1 day before and 1 day after vaccination (38). Serum was collected every 2 weeks post-vaccination for analysis of antibody responses (Fig. 3a). Despite the interferon antibody blockade, mice exhibited no significant weight loss or signs of illness over the course of the study, even with the highest dose of vaccine (10^8^ PFU/mouse) (Fig. 3b).

To characterize the humoral response to rVSV-MACV vaccination, we measured MACV GPC-specific IgG antibody titers via enzyme-linked immunosorbent assay (ELISA). Plates were coated with purified, soluble, recombinant MACV GPC. Serum from rVSV-EBOV- and PBS-immunized mice did not bind and served as negative controls. Both vaccines elicited a robust IgG response by day 14 after vaccination, with MACV-specific antibody titers in all groups reaching around 10^4^ (Fig. 3c). We did not observe significant differences between responses to rVSV-MACV5 and rVSV-MACV1 following the initial vaccination. The titers were not increased significantly upon boost for either vaccine. We observed a significant drop in antibody titers for the groups receiving 10^6^ and 10^7^ PFU of rVSV-MACV5 on day 56; however, this trend was not repeated in subsequent experiments. Overall, at these vaccine doses, we could not observe significant differences between antibody titers in serum from rVSV-MACV5-vaccinated mice compared to rVSV-MACV1-vaccinated mice.

To address whether the high vaccine doses used in these initial experiments were masking differences in the immunogenicity of the two rVSV vaccines, we reduced the dose of the vaccines. C57BL/6 mice were vaccinated with 10^3^, 10^4^, or 10^5^ PFU/mouse as described. Serum samples were analyzed via ELISA for total IgG as before. In contrast to the results with higher vaccine doses, here we observed a dose-dependent response to vaccination. Additionally, rVSV-MACV5 elicited higher MACV GP-specific IgG titers than rVSV-MACV1 at all time points. The differences between rVSV-MACV5 and rVSV-MACV1 were especially apparent and significant at the lowest dose (10^3^ PFU). As seen previously with the higher vaccine doses, a booster immunization did not significantly increase the antibody titers (Fig. 3e).

To investigate the function of the MACV GP-specific antibodies, we measured neutralizing antibody titers via focus reduction neutralization test of 50% (FRNT_50_). At the higher doses (10^6^–10^8^ PFU/mouse), three mice immunized with rVSV-MACV5 had developed neutralizing antibodies at day 14 post-vaccination. In contrast, none of the mice immunized with rVSV-MACV1 developed detectable neutralizing antibodies by day 14 post-vaccination. The neutralizing titer in all groups continued to increase from day 14 to day 26 post-vaccination. Although the neutralizing antibody response to rVSV-MACV5 did not appear to be boosted, neutralizing responses to rVSV-MACV1 did increase after the second dose of vaccine (Fig. 3d). Relative to neutralizing titers against other viral antigens, overall neutralizing titers were low for all groups, with peak titers between 128 and 256.

In a manner similar to the IgG responses, the analysis of neutralizing responses in mice receiving lower vaccine doses showed a more significant distinction between the vaccination groups, with rVSV-MACV5 consistently inducing significantly higher levels of neutralizing antibodies compared to rVSV-MACV1 (Fig. 3f). At these lower doses, rVSV-MACV1 failed to induce neutralizing antibodies in most mice, and only mice immunized with two doses of 10^5^ PFU rVSV-MACV1 developed neutralizing antibodies, albeit at low levels. Additionally, we noted a positive correlation between total IgG titers and neutralizing titers for animals immunized with 10^7^ PFU/mouse (Fig. 3g).

To evaluate the durability of the humoral immune response generated by rVSV-MACV vaccination, serum from immunized mice was sequentially sampled over time until day 118 (approximately 4 months) after the initial immunization. There was no significant decline in total IgG titers in mice immunized with a prime and boost of the lowest dose (10^3^ PFU) of either vaccine (Fig. 3h). The neutralizing antibody response to rVSV-MACV5 did not decline either. At this dose, the neutralizing antibody response to rVSV-MACV1 was undetectable and did not further develop with time (Fig. 3i).

Lastly, we also evaluated the humoral immune response to a single dose of 10^7^ PFU of either rVSV-MACV5 or rVSV-MACV1. A single dose of either vaccine elicited MACV-specific antibodies as expected from previous experiments (Fig. 3c and d). Total IgG titers were constant for months after immunization and did not decline, with rVSV-MACV5 inducing higher levels than rVSV-MACV1 (Fig. 3j). A single dose of rVSV-MACV5 did elicit neutralizing antibodies that developed over time with a significant increase from day 14 to day 42. The neutralizing antibody response to rVSV-MACV1 was undetectable and did not increase over the course of the study (Fig. 3k). Overall, these results indicate that rVSV-MACV5 induces higher levels of total MACV GPC-specific IgG and neutralizing antibodies, and that a single dose of rVSV-MACV5 elicits a robust and durable humoral immune response.

### Breadth of the humoral immune response to vaccination

**Fig 4:**
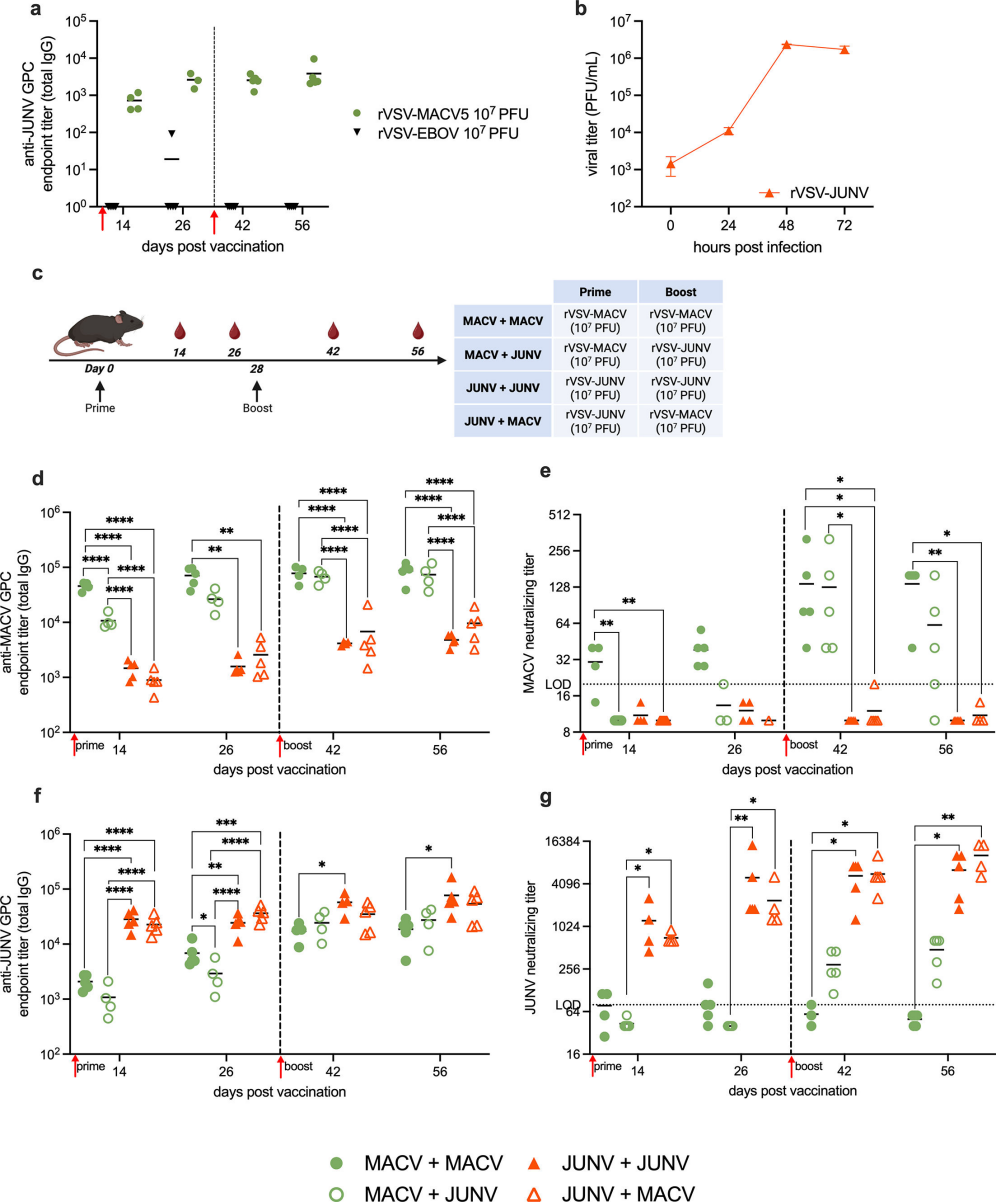
Antibody response to immunization with rVSV-MACV5 and rVSV-JUNV. (**a**) Total JUNV GPC-specific IgG titers in serum from mice (*n* = 5) vaccinated with 10^7^ PFU of rVSV-MACV5 or rVSV-EBOV. (**b**) Growth kinetics of rVSV-JUNV in Vero E6 cells infected at an MOI of 0.0001. Supernatants were collected every 12 hours, and viral titer was evaluated via plaque assay. Data show mean ± standard deviation. (**c**) Immunization timeline in C57BL/6 mice. Mice were vaccinated on day 0 and boosted 28 days later with the same dose. The table outlines which vaccine was administered as prime or boost in each group. To allow for VSV replication, on days −1 and +1 relative to vaccination, an anti-interferon-alpha/beta receptor (anti-IFNAR) blocking antibody (0.5 μg/mouse) was administered. The timing of blood collection is indicated by red droplets, which took place every 2 weeks for serological analysis of antibody titers. Panel a was created using BioRender.com. Total MACV-specific IgG titers (**d**) and neutralizing titers (**e**) in serum from vaccinated mice. Total JUNV-specific IgG titers (**f**) and neutralizing titers (**g**) in serum from vaccinated mice. Titers from individual animals are indicated by individual symbols, and the horizontal line indicates the geometric mean of the group. Homologous prime-boost vaccination is indicated by solid shapes, whereas heterologous prime-boost is indicated by open shapes. Each point represents the mean (for endpoint titer/ELISA) or geometric mean (for neutralizing titer/FRNT) from 2 to 3 independent ELISA or FRNT analyses of each serum sample. Statistical analysis was performed to compare different vaccination groups on individual days. When data met the assumption of normality, one-way ANOVA with Tukey’s multiple comparisons test was utilized. The Kruskal–Wallis with Dunn’s multiple comparisons test was used for non-normal data, *P < 0.05, **P < 0.01, ***P < 0.001, ****P < 0.0001. Lack of an illustrated comparison bar between comparison groups indicates no significance between groups, where *P* > 0.05. LOD indicates the limit of detection for the assay.

Because rVSV-MACV5 elicited higher antibody titers than rVSV-MACV1, we chose to move forward with this vector in subsequent experiments. To explore whether the rVSV-MACV5 vaccine could elicit cross-reactive antibodies against other NW arenaviruses, we used a JUNV GPC ELISA to measure JUNV GPC-specific cross-reactive antibodies. JUNV is the most closely related NW arenavirus to MACV (39). The JUNV and MACV GPCs share 69% amino acid identity and 79.4% similarity. JUNV-specific IgG antibodies were measured in serum from mice vaccinated with 10^7^ PFU rVSV-MACV5. The IgG titers initially measured around 10^3^ at day 14 post-vaccination and rose about half a log by day 26 (Fig. 4a). Junin-specific IgG titer did not increase upon receipt of a booster dose of rVSV-MACV5. We included serum from rVSV-EBOV-immunized mice as a negative control, which showed no anti-JUNV GPC binding, except for one sample from day 26 that had a minimal background signal.

To further explore this idea of a broadly applicable mammarenavirus vaccine, we sought to develop a vaccine regimen that could elicit higher levels of cross-reactive antibodies. With other viruses, sequential immunization with related antigens has been shown to induce broadly reactive antibody responses (40–42). We hypothesized that administering a heterologous antigen prime-boost vaccination using both rVSV-MACV5 and rVSV-JUNV might elicit higher levels of broadly reactive antibodies. The rVSV-JUNV we used is identical to rVSV-MACV5, except it expresses the JUNV GPC in place of MACV GPC. rVSV-JUNV grows well and replicates to high titers (between 10^6^ and 10^7^ PFU/mL) in Vero E6 cells, slightly lower than the peak titer of rVSV-MACV5 (Fig. 4b). Previous studies established that a single dose of rVSV-JUNV elicits a protective immune response against lethal JUNV challenge in guinea pigs (43).

To test whether our heterologous antigen prime-boost regimen could induce a humoral immune response with greater breadth, we evaluated rVSV-MACV5 compared to and in combination with rVSV-JUNV. We immunized groups of mice as follows: rVSV-MACV5 then rVSV-MACV5, rVSV-MACV5 then rVSV-JUNV, rVSV-JUNV then rVSV-JUNV, and rVSV-JUNV then rVSV-MACV5, where the first vaccine indicated was given as the prime and the second as the boost. The boost was given 28 days after the initial immunization (Fig. 4c). All vaccines were 10^7^ PFU delivered i.p., with 0.5 μg of an anti-IFNAR blocking antibody administered 1 day before and 1 day after vaccination.

To characterize the humoral response to vaccination, we measured MACV or JUNV GPC-specific IgG antibody titers via ELISA as described previously. Plates were coated with purified soluble recombinant MACV GPC or JUNV GPC. As previously observed, animals immunized with two doses of rVSV-MACV5 (closed green circles) had high levels of anti-MACV GPC antibodies, reaching almost 10^5^ by day 14 post-vaccination. Titers were not boosted upon receipt of a second dose (Fig. 4d). Mice from this group exhibited low levels of anti-JUNV GPC cross-reactive antibodies, with titers around 10^3^ (see Fig. 4a), which were boosted about 1 log following receipt of the second dose (Fig. 4f). The antibody response in mice that received a heterologous boost (open green circles, boosted with rVSV-JUNV instead of rVSV-MACV5) was remarkably similar to the homologous boost (Fig. 4d and f). Specifically, in mice primed with rVSV-MACV5, immunization with rVSV-JUNV as the boost did not increase the anti-JUNV GPC titers significantly compared to a homologous boost (Fig. 4e).

In contrast, animals immunized with two doses of rVSV-JUNV (closed orange triangles) had high levels of anti-JUNV GPC antibodies (titer around 10^4.5^) that remained constant following the second dose (Fig. 4f). Serum from these animals also had low levels of cross-reactive anti-MACV GPC antibodies, which were only boosted about half a log following the second dose (Fig. 4d). Again, a heterologous boost (open orange triangles) elicited an antibody response that was not significantly different than the homologous boost (Fig. 4d and f). In mice that received an rVSV-JUNV prime, the heterologous boost (rVSV-MACV5) did not increase the MACV GPC-specific antibody titer (Fig. 4d).

Overall, immunization with rVSV-MACV5 as the prime, regardless of the boost, achieved the highest level of both MACV and JUNV GPC-specific antibodies (Fig. 4d and f). Surprisingly, immunization with two doses of rVSV-MACV5 elicited anti-JUNV GPC antibodies at a level comparable to two doses of rVSV-JUNV, with no significant difference between any of the four vaccine groups (Fig. 4f). Immunization with rVSV-JUNV as the prime did not elicit high levels of MACV GPC-specific antibodies, regardless of whether rVSV-MACV5 was used as the boost.

To further characterize the humoral immune response, we also measured MACV and JUNV neutralizing antibody titers via FRNT. Immunization with two doses of rVSV-MACV5 (closed green circles) elicits cross-reactive but not cross-neutralizing antibodies (Fig. 4g). However, a heterologous boost involving rVSV-MACV5 prime followed by rVSV-JUNV boost (open green circles) does induce low levels of JUNV-neutralizing antibodies (Fig. 4g). Immunization with rVSV-JUNV never elicits cross-neutralizing antibodies, regardless of which boost (homologous or heterologous) was used (Fig. 4e). It is striking that despite the presence of cross-reactive antibodies to MACV GPC, we do not observe MACV cross-neutralizing antibodies.

It is also interesting that there is a non-reciprocal relationship between MACV and JUNV. Priming with rVSV-MACV5 does result in cross-reactive and cross-neutralizing antibodies when a heterologous boost is used, whereas priming with rVSV-JUNV does not allow for the development of cross-neutralizing antibodies, and also results in lower overall titers of cross-reactive antibodies, even when the heterologous rVSV-MACV5 is used as the boost. Together, these data suggest that rVSV-MACV5 could be useful in eliciting broadly reactive antibodies to multiple NW arenaviruses.

### Cellular immune response to rVSV-MACV vaccines in mice

VSV vaccines are valued for their ability to elicit a robust, balanced humoral and cellular immune response (44, 45). The role of cellular immunity in controlling MACV and other NW arenavirus infections is relatively understudied, and there is relatively little published on cellular responses to MACV vaccination. To characterize the cellular response to rVSV-MACV vaccination, we immunized mice with a single 10^4^ PFU dose of rVSV-MACV5 or rVSV-MACV1. On day 10 after immunization, splenocytes were isolated from immunized mice to analyze T-cell responses by enzyme-linked immunosorbent spot (ELISpot) assays. Splenocytes were stimulated with a peptide pool of 49 peptides that spans MACV GP1 or a single VSV N peptide overnight, and IFN-γ secretion was measured to evaluate T-cell activation. MACV GP1 is a component of MACV GPC; GP1 is the surface glycoprotein responsible for receptor binding (6).

**Fig 5:**
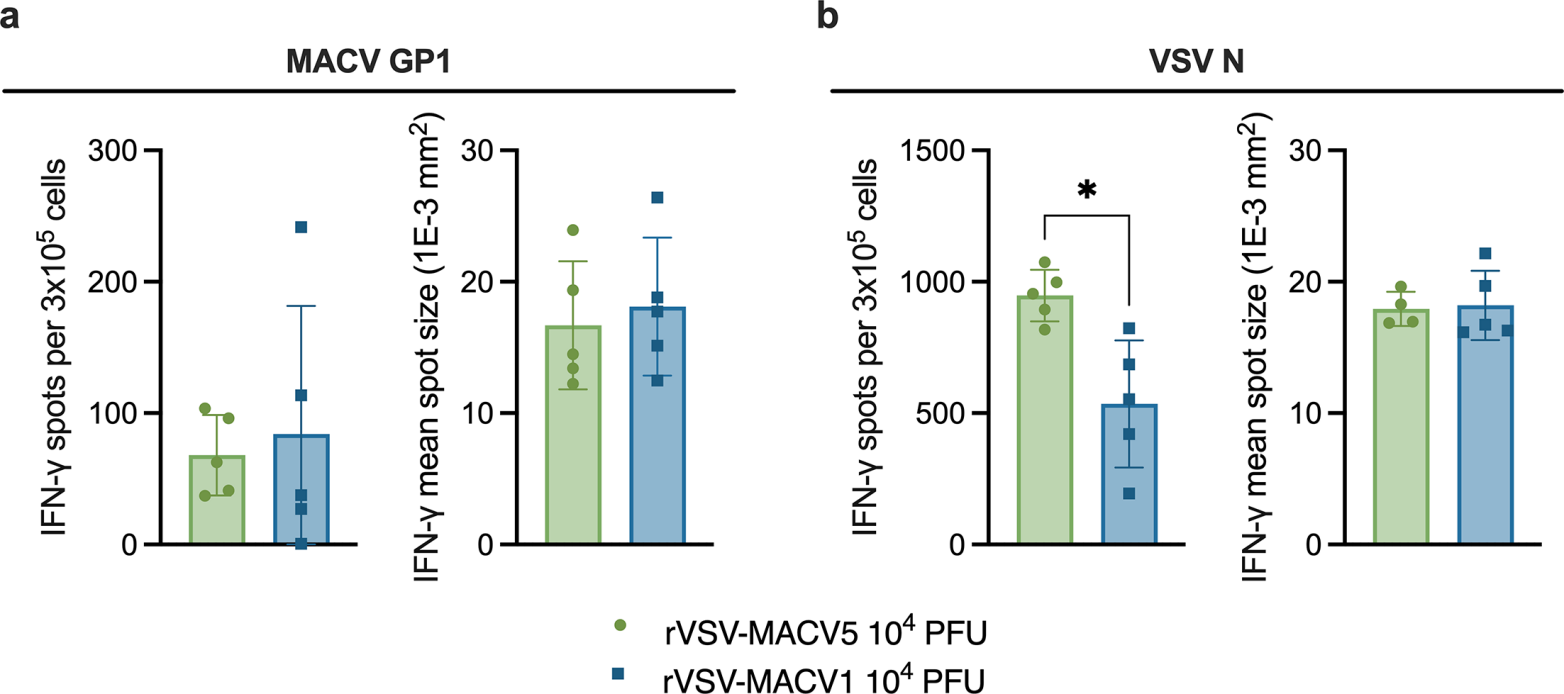
rVSV-MACV5 and rVSV-MACV1 induce T-cell responses in mice. ELISpot assay data for MACV GP1-specific (**a**) and VSV N-specific (**b**) IFN-γ responses of splenocytes from C57BL/6 mice (*n* = 5 per group) that were immunized with 10^4^ PFU rVSV-MACV5 (green circles) or rVSV-MACV1 (blue squares). Data points denote spots per 3 x 10^5^ cells (left) and mean spot sizes (right) and represent the mean of two technical replicates per animal. The mean of the group is shown by the bar height, and error bars indicate ±SD. Welch’s t test was performed; significant differences between groups linked by brackets are indicated by an asterisk: *P < 0.05. Lack of an illustrated comparison bar between comparison groups indicates no significance between groups, where *P* > 0.05.

rVSV-MACV5 and rVSV-MACV1 both induced IFN-γ responses when splenocytes were stimulated with MACV GP1, indicating the presence of MACV GPC-specific T cells (Fig. 5a). There were no significant differences between T-cell responses to immunization with rVSV-MACV5 (average spot count: 68) and rVSV-MACV1 (average spot count: 84). When splenocytes were stimulated with a VSV N peptide, rVSV-MACV5-vaccinated mice had a significantly higher level of reactive T cells (average spot count: 947.8) than mice immunized with rVSV-MACV1 (average spot count: 535) (Fig. 5b). These data suggest that there is higher *in vivo* replication for rVSV-MACV5 than for rVSV-MACV1. Furthermore, the IFN-γ spot count is much higher when splenocytes are stimulated with the VSV N peptide than when the MACV GP1 peptide pool is used. Overall, the T-cell analysis shows that both rVSV-MACV vaccine constructs induce T-cell responses.

## DISCUSSION

MACV is a significant public health threat for which there are currently no approved vaccines. In an effort to engineer an optimized vaccine candidate, we created and evaluated two different recombinant VSV vaccine constructs expressing the MACV GPC in different genome positions (rVSV-MAV5 and rVSV-MACV1). In this study, we show that both vaccines elicit MACV GPC-specific antibodies and neutralizing antibodies in mice, as well as MACV GPC-specific T-cell responses, with rVSV-MACV5 inducing higher overall antibody titers and higher levels of neutralizing antibodies. Beyond potential applications for MACV, direct comparison of the rVSVs expressing MACV GPC in different genome positions provides insight into rVSV as a vaccine vector that may be considered when designing rVSV vaccines for other pathogens.

rVSV is a powerful platform for vaccine development, and further understanding of how genome rearrangements impact rVSV vaccines is important. Both rVSV-MACV viruses were launched and rescued relatively easily, and both grow well in cell culture. However, rVSV-MACV5 does grow to slightly higher titers than rVSV-MACV1 (Fig. 1b), as would be expected for a virus construct with a genome that is more similar to the wild-type gene order. VSV gene expression follows a transcriptional gradient, with higher expression of genes at the 3′ end of the genome, and an approximately 30% decrease in each subsequent gene (46). Our data comparing protein expression in rVSV-MACV5 and rVSV-MACV1 reflect this, as rVSV-MACV1 expressed higher levels of MACV GPC than rVSV-MACV5 in both infected cells and on purified virions.

However, this increased amount of antigen did not translate to an improved immune response. rVSV-MACV5 induced higher levels of total IgG antibodies than rVSV-MACV1. Our results were unexpected; previously published data comparing VSV genome rearrangements showed that a VSV expressing its native glycoprotein G in position 1 (upstream of N) elicited higher levels of antibodies than wild-type VSV with G in position 4 (between M and L) (34). However, as mentioned previously, the rearrangement of the VSV genome in this case shifted all other VSV genes relative to wild-type VSV G and attenuated the resultant viruses; therefore, there are other factors, such as expression levels of N, P, M, and L, that may influence the result. Other studies evaluated the cellular immune response and protective efficacy of rVSVs expressing the EBOV GP in different genome positions (47). The rVSV expressing EBOV GP in genome position 1 did elicit higher levels of EBOV GP-specific cellular and humoral immune responses than when EBOV GP was expressed in the third or sixth positions. Again, the movement of EBOV GP to position 1 shifted the position of other VSV genes, and direct evaluation of the position of EBOV GP in the genome was not assessed. In contrast, we added an additional ORF expressing eGFP to the rVSV-MACV genomes to maintain the genome position of the VSV genes (N, P, M, and L) relative to each other (Fig. 1a). Therefore, in this study, we directly compare the effect of altering MACV GPC position in the VSV genome on humoral and cellular immune responses to vaccination. These findings have implications for rVSV vaccine design as genome rearrangements can impact attenuation of the viral vector as well as the immune response to the vaccine. Additionally, the data from our study support the idea that the rVSV vaccine elicited immune responses to different viral antigens that are antigen-specific. Therefore, it is important that vaccine candidates for each pathogen be evaluated empirically.

One possible explanation for the superior immune response of rVSV-MACV5 could be due to the differences in viral replication; although both viruses exhibited similar replication kinetics *in vitro*, rVSV-MACV5 consistently reached titers that were about half a log higher than rVSV-MACV1 (Fig. 1b). Furthermore, our T-cell analysis showed that rVSV-MACV5 induced higher levels of VSV-N reactive T cells than rVSV-MACV1, suggesting that rVSV-MACV5 replicates more effectively *in vivo* as well. We attempted to directly measure viral replication *in vivo* via qPCR on RNA from the blood of immunized animals. Other labs have reported detection of rVSVs in the blood of immunocompromised mice, such as C57BL/6 IFNAR^−/−^ mice (48). In contrast, we used immunocompetent C57BL/6 mice with the administration of an anti-IFNAR antibody on day −1 and day 1 post-immunization to induce transient immunosuppression. However, we were unable to detect circulating virus in the blood of animals immunized with either rVSV-MACV5 or rVSV-MACV1. We were also unable to detect circulating rVSV-EBOV in control animals, even when mice received the highest dose (10^8^ PFU/mouse). rVSV-EBOV is lethal in IFNAR^−/−^ mice (49), but it does not cause any illness or mortality in mice treated with the anti-IFNAR blocking antibody, indicating that in our model, the innate immune system may be diminished but not completely abrogated. These results indicate that in spite of the interferon receptor blockade employed, rVSV replication may still be controlled by the innate immune system to some degree. Future work will investigate whether differences in viral replication between rVSV-MACV5 and rVSV-MACV1 are responsible for the differences in humoral and cellular immune responses.

Furthermore, we anticipated poor rVSV-MACV replication in C57BL/6 mice due to a receptor–glycoprotein mismatch. MACV GPC does not efficiently bind the laboratory mouse ortholog of the main MACV receptor, transferrin receptor 1 (TfR1) (50). Therefore, MACV must enter via a less efficient pathway (51). Thus, we used the anti-IFNAR blocking antibody to induce transient immunosuppression to allow for more rVSV replication. In preliminary studies, we found that the use of the anti-IFNAR antibody blockade was not necessary for the induction of total IgG or neutralizing antibodies, but its use allowed us to achieve slightly higher antibody titers and observe larger differences between vaccination groups.

The use of a transient immunosuppression model has important implications for future challenge studies. The gold standard small animal model for MACV challenge is the outbred Hartley guinea pig. MACV GPC binds to and utilizes the guinea pig ortholog of TfR1 for viral entry (52). Therefore, in the guinea pig model, we would anticipate robust rVSV-MACV replication without any transient immunosuppression such as the anti-IFNAR antibody blockade. In guinea pig challenge studies, it will be important to evaluate and compare replication of rVSV-MACV5 and rVSV-MACV1, as well as the antibody response to each, to determine whether our observations hold true in immunocompetent animal models.

A single dose of either rVSV-MACV5 or rVSV-MACV1 elicited high antibody titers, which, in general, were not boosted significantly upon receipt of a second dose (Fig. 3c). In contrast, previous studies with rVSV vaccines did note a significant boost in antibody titers after a booster immunization. For example, in the study investigating humoral immune responses to rVSV-EBOV with EBOV GP in genome position 1, a second dose of vaccine did boost total EBOV GP-specific ELISA titers from 10^3.5^ to 10^5^ (47). In another study that tested an rVSV-JUNV vaccine, the JUNV GPC-specific IgG titers were significantly higher in guinea pigs that received two doses of 10^7^ PFU than in mice that received a single dose of 10^7^ PFU (43). It is possible that the development of neutralizing antibodies by the time the boost was administered on day 28 quickly controls viral replication and dampens the effect of the second dose. These data indicate that a single-dose vaccine regimen may be sufficient. This is especially important in lower-resource areas and outbreak settings where a single dose would be more efficient and accessible. The only rVSV vaccine currently approved by the FDA, rVSV-EBOV, is given as a single dose (53).

Neutralizing antibodies are thought to be a correlate of protection for NW arenaviruses. One of the only successful treatments for NW arenavirus infection is convalescent plasma transfer, although this has only been definitively shown for JUNV. In a double-blind placebo-controlled study, transfer of immune plasma into infected patients reduced the case-fatality of JUNV from 16.5% to 1.1% (54). Survival correlated with neutralizing antibody titer in the serum (55). Therefore, it is thought that antibodies are important in controlling infection. Convalescent serum transfer as a treatment for MACV has been investigated in animal models; anti-MACV GPC sera are sufficient for protection from lethal challenge in the established Hartley guinea pig model (56). However, controlled clinical studies for MACV have not been reported. Historically, serum transfer has been used sporadically to treat cases of MACV infection, although there is no standardized dose or protocol, and the limited availability of convalescent serum makes further investigation difficult (57, 58). In the present study, rVSV-MACV5 elicited higher levels of neutralizing antibodies than rVSV-MACV1. Interestingly, the initial neutralizing titers to all vaccinations were low, but titers increased from day 14 post-vaccination to day 26 post-vaccination. It is unclear why the neutralizing response continued to develop over time when it is believed that the rVSVs should be quickly cleared by the immune system. Overall, MACV neutralizing titers were quite low, rarely exceeding a FRNT_50_ reciprocal dilution value of 256 (Fig. 3d). Our observations are similar to studies of the OW arenavirus LASV; an rVSV expressing the LASV GPC induced IgG and neutralizing antibodies by day 26 post-vaccination in non-human primates. As was the case with MACV, the neutralizing antibodies were relatively low compared to the IgG titer (59). Furthermore, an early report from 1969 finds that neutralizing titers (reported as PRNT80 reciprocal dilution values) in serum from MACV-infected patients peaked at 128 at 100 days after infection (60). These neutralizing titers are much lower than those seen when compared to rVSV vaccines with other viral antigens. For example, in mice immunized with an rVSV vaccine expressing the glycoprotein from the bunyavirus Severe fever with thrombocytopenia syndrome virus (SFTSV), the neutralizing titers were between 200 (post-prime) and 1,280 (post-boost) (61). It seems possible that the overall magnitude of neutralizing antibody response to arenaviral glycoproteins is not high compared to other viruses. Moreover, this may be an inherent feature of arenaviruses, and low neutralizing antibody levels may be sufficient for protection.

We also investigated the breadth of the humoral response to rVSV-MACV5. JUNV is the most closely related NW arenavirus to MACV (39), with the JUNV and MACV GPCs sharing 69% amino acid identity and 79.4% similarity. rVSV-MACV5 induced cross-reactive JUNV-GPC-specific antibodies (Fig. 4a). Furthermore, it has been shown that sequential immunization with related antigens can induce broadly reactive antibody responses (40–42). When comparing rVSV-MACV5 and rVSV-JUNV and combining them into a heterologous antigen prime-boost regimen, we observed a non-reciprocal relationship between MACV and JUNV. Priming with rVSV-MACV makes it possible to induce a broader immune response, including JUNV GPC-specific antibodies with titers that are comparable to immunizing with rVSV-JUNV alone (Fig. 4f). Additionally, an rVSV-MACV5 prime followed by an rVSV-JUNV boost elicited cross-neutralizing antibodies (Fig. 4g). In contrast, priming with rVSV-JUNV never resulted in high cross-reactive or cross-neutralizing antibodies, regardless of if rVSV-MACV5 was used as a boost (Fig. 4e and g).

One striking difference that we noticed was in the magnitude of neutralizing titers against MACV and JUNV. As mentioned previously, the neutralizing titers to MACV are extremely low, peaking at around 256 reciprocal dilution FRNT_50_ value. However, the JUNV neutralizing titers peak at around 4,096 (Fig. 4g). Although these viruses are closely related, there are notable differences in the structure of their GPCs, which may account for this non-reciprocal relationship. Previous studies investigating cross-reactive antibodies between MACV and JUNV have primarily focused on whether JUNV GPC-elicited antibodies could cross-react with MACV GPC (62, 63). Some anti-JUNV antibodies were identified that cross-react with MACV GPC to varying degrees (64). However, other studies found that structural differences, namely the presence of a unique loop called loop 10 in MACV GPC, interfere with the binding of cross-reactive antibodies (65). Further investigation of the breadth of the immune response to rVSV-MACV vaccination would elucidate whether rVSV-MACV may be broadly applicable against other NW arenaviruses. Additionally, cross-protection studies in the Hartley guinea pig model would show whether the levels of cross-reactive antibodies are sufficient for protection from JUNV or other related NW arenaviruses.

The rVSV-MACV vaccines elicited robust humoral immune responses, but we acknowledge that characterization of the immune response does not translate directly to protection. Therefore, future studies should test the protective efficacy of these vaccines in the established Hartley guinea pig lethal MACV challenge model. Given that antibodies have been correlated with protection for the NW arenaviruses, we anticipate successful protection from challenge.

Vaccine development must consider the balance of humoral and cellular responses to vaccination. Although the role of cellular immunity is less studied for MACV infection, it was important to evaluate whether our vaccine candidates induce MACV GPC-specific T-cell responses. MACV GP1 is a component of MACV GPC; GP1 is the surface glycoprotein responsible for receptor binding (6). Both rVSV-MACV5 and rVSV-MACV1 induced MACV GP1-specific T-cell responses, but there was no significant difference between vaccine groups. It is possible that increased replication of rVSV-MACV5 and the increased MACV GPC antigen production of rVSV-MACV1 balance out and obscure the differences between MACV GPC-specific T-cell responses to each vaccine. rVSV-MACV5 induced significantly higher levels of VSV N-specific T cells than rVSV-MACV1. As mentioned previously, this is likely another indicator that the improved replication of rVSV-MACV5 plays a role in stimulating a more robust humoral immune response in mice.

In this study, we characterize for the first time recombinant VSV vaccines for MACV. We utilized rVSVs expressing MACV GPC in different genome positions to directly compare the effect of gene order rearrangements on humoral and cellular immune responses. As we and others have shown, genome rearrangements can impact viral attenuation and immunogenicity, but the effects are antigen-specific. Here, we show that both rVSV-MACV5 and rVSV-MACV1 are well tolerated and immunogenic in mice, with rVSV-MACV5 inducing higher humoral immune responses. Our data suggest that a single dose of rVSV-MACV5 effectively induces a robust cellular and humoral immune response, including cross-reactive antibodies that may be broadly applicable to other NW arenaviruses. These findings support further investigation of rVSV-MACV5 as a possible vaccine candidate for MACV.

## MATERIALS AND METHODS

### Cell lines and plasmids

ATCC verified and mycoplasma-free 293T and Vero E6 cells were maintained in DMEM (Corning, #10-013-CV) containing 10% CCS (HyClone, #SH30087.03). Cells were passaged every 2–3 days.

The pVSV eGFP RABVG plasmid (Addgene) was used as the template for creating vectors expressing the VSV genome and MACV GPC or JUNV GPC. The full-length, codon-optimized MACV GPC sequence (GenBank accession number: Q6IUF7.1) and JUNV GPC sequence (GenBank accession number: P26313.2) were obtained as gBlocks (Integrated DNA Technologies, Coralville, Iowa) and cloned into pCG1 expression vectors to create pCG1 MACV GPC or pCG1 JUNV GPC. rVSV-MACV5 was generated by inserting MACV GPC into the pVSV eGFP RABV G backbone between M and L, replacing RABV-G. rVSV-JUNV was created in the same way. rVSV-MACV1 was generated by inserting MACV GPC upstream of N in pVSV eGFP RABV-G and moving eGFP to the site between M and L. All cloning was carried out using restriction cloning; restriction sites were introduced to the sequences using the following primer pairs. Restriction sites were added to MACV GPC using MACV MluForward, CTGTTTACGCGTCATGGGTCAACTGATTAGC, and MACV Eag reverse, GCTAGCGCGGCCGCCTAATGTCTCTTATGCCAAAT. The Xho site was removed from MACV (mutate CTCGAG to CTCGAA) using MACV CTCGAA Forward, TGTCTCGAAGAATGGATGCTGATAGCGGC, and MACV CTCGAA Reverse, CCATTCTTCGAGACAATATCCACCGGGC. Xho1 + Msc1 was added to MACV GPC using MACV Xho1 Forward, AATCAGAATTCTCGAGATGGGTCAACTGATTAGCTTTT, and MACV Msc1 Reverse, GTTTTTTTCATATGGCCACTAATGTCTCTTATGCCAAATTGTT. Mlu1 + Not1 was added to eGFP from RABV using eGFP Mlu1 Forward, TTTCCTTGACACGCGTATGGTGAGCAAGGGCGAGG, and eGFP Not1 Reverse, TTGTGCAGGGCGGCCGCTTGTACAGCTCGTCCATGCC. The pVSV-JUNV genome was generated similarly. After generation, vectors were sequenced by the Eurofins Complete Plasmid Sequencing Service.

VSV launch plasmids were also obtained from Addgene. pVSV eGFP RABV-G was a gift from Connie Cepko (66) (Addgene plasmid #31833; https://www.addgene.org/31833/; RRID:Addgene_31833), pCAG-VSVN was a gift from Ian Wickersham (Addgene plasmid #64087; https://www.addgene.org/64087/; RRID:Addgene_64087). pCAG-VSVP was a gift from Ian Wickersham (Addgene plasmid #64088; https://www.addgene.org/64088/; RRID:Addgene_64088). pCAG-VSVL was a gift from Ian Wickersham (Addgene plasmid #64085; https://www.addgene.org/64085/; RRID:Addgene_64085). T7opt in pCAGGS was a gift from Benhur Lee (67) (Addgene plasmid #65974; https://www.addgene.org/65974/; RRID:Addgene_65974).

### Launching recombinant VSV

Recombinant VSV viruses expressing an additional open reading frame encoding eGFP were launched as described previously (35). Briefly, 293T cells were plated 24 hours before transfection with Lipofectamine 3000 (Thermo Fisher Scientific, Rockford, IL, USA). The plasmids transfected were VSV genome, VSV N, VSV P, VSV L, and T7 opt polymerase at a 1:3:1:1:2 molar ratio with a total of 2.7 μg DNA per well in a six-well plate.

Media was changed 18–24 hours post-transfection. Cells were observed for eGFP expression and cytopathic effects, including cell rounding and death. Virus was collected 2–4 days post-transfection, cell debris was spun down at 1,250 × *g* for 10 minutes, then supernatant was passaged onto a monolayer of Vero E6 cells.

All recombinant virus stocks were grown in Vero E6 cells by infecting a confluent 15 cm plate at an MOI of 0.0001. Virus was collected 48 hours post-infection, and media was clarified by centrifugation at 1,250 × *g* for 10 min at 4°C. Virus was then frozen at −80°C until concentrated via ultracentrifugation. Virus was spun on a 20% sucrose cushion for 2 hours at 28,000 rpm in an Optima XPN-80 ultracentrifuge (Beckman Coulter, Indianapolis, IN, USA) with an SW 32 Ti rotor. Virus pellet was resuspended in PBS, aliquoted, and stored at −80°C.

### Plaque assay

Titers of recombinant VSV viruses were determined via plaque assay. Vero E6 cells were plated in a six-well plate 24 hours prior to the assay. Viral stocks were serially diluted and transferred onto confluent Vero E6 cells, then incubated at 37°C + 5% CO_2_ for 1 hour. Virus was removed, cells washed with PBS, and covered with 2.5% Avicel (Millipore Sigma) and DMEM + 10% CCS. After 2 days of incubation at 37°C with 5% CO_2_, 4% paraformaldehyde (PFA) was added to the wells to fix cells. Wells were rinsed, stained with crystal violet, and washed with water to remove excess stain. Plaques were counted, and viral titer was calculated as PFU/mL of original stock solution.

### SDS-PAGE and western blot

Vero E6 cells were infected with rVSV-MACV5 and rVSV-MACV1 at an MOI of 0.0001. After 48 hours of infection, cells were collected from the plate, pelleted by centrifugation at 1,250 × *g* for 10 minutes, then washed 1× with PBS. The cell pellet was resuspended in RIPA buffer + cOmplete protease inhibitor (Roche) and rocked at 4°C for 1 hour. The samples were spun at 21300 × *g* at 4°C for 10 minutes, and the supernatant was collected as cell lysate. Cell lysate from infected cells and purified rVSV virions were mixed with 6× loading dye and boiled at 95°C for 5 minutes. Viral proteins were examined using SDS-PAGE. For Western blotting, viral proteins were separated and transferred onto a poly (vinylidene fluoride) (PVDF) membrane, blocked with blocking buffer (5% NFDM in TBS + 0.5% Tween 20), and incubated with serum from MACV mRNA-immunized mice (1:1,000 in blocking buffer) at 4°C overnight on a plate rocker. This was followed by incubation for 1 hour with anti-mouse HRP-conjugated secondary antibody, the addition of substrate (Invitrogen, #31430), and imaging on an Amersham Imager 600 (GE Healthcare, Chicago, IL, USA).

### Animal care and use

For mouse studies, 8-week-old female C57BL/6 mice (JAX, stock #000664) were used. Mice were maintained according to the *Guide for the Care and Use of Laboratory Animals*. All procedures were performed on animals after they were anesthetized by trained personnel. All efforts were made to minimize the pain and suffering of animals.

### Immunization and blood collection

Viruses were diluted in sterile PBS just prior to inoculation. Eight-week-old female C57BL/6 mice (JAX, stock #000664) were anesthetized with isoflurane and immunized i.p. with rVSV (150 µL total volume). To allow efficient VSV replication, 0.5 µg/mouse of an anti-interferon-alpha/beta receptor (anti-IFNAR-1) blocking antibody (Clone MAR1-5A3) (Leinco Technologies Inc, #I-1188 or BioLegend, #127321) was administered 1 day before and 1 day after vaccination. Blood was collected via the submandibular route using Goldenrod lancets 5 mm (Medipoint, Mineola, NY, USA). Serum was separated from blood by centrifugation at 8,000 rpm for 30 minutes at 4°C in an Eppendorf 5424R centrifuge (Eppendorf, Enfield, CT, USA). EDTA was added to each sample for a final concentration of 5 mM. Serum was heat-inactivated by incubating at 53°C for 30 minutes, then centrifuged at 15,000 rpm for 20 minutes. Serum samples were stored at −20°C.

### Production of recombinant proteins

MACV GPC or JUNV GPC sequence was cloned from pCG1 MACV GPC or pCG1 JUNV GPC plasmids, respectively, into a Sport6 expression vector. This vector includes a trimerization domain and a 6His tag. Expi293 cells (Gibco, #A14527) grown in Expi293 Expression media (Gibco, #A1435101) were transfected with the Sport6 plasmid using PEI Max (Kyfora Bio, 24765). Supernatant was collected 4 days post-transfection and purified by nickel-nitrilotriacetic acid resin (Qiagen, #30210) according to the manufacturer’s protocol. Eluted protein was concentrated and buffer exchanged into PBS using an Amicon Ultra-15 Centrifugal Filter with a 30 kDa MWCO (Millipore, #UFC903024). Protein was evaluated by SDS-PAGE followed by Coomassie staining or Western blot using an anti-His antibody to detect both MACV and JUNV proteins. In both the gel and the blot, a single band for each protein was observed. Purified protein was quantified using Pierce BCA protein assay kit (Thermo Scientific, 23225), then aliquoted and stored at −80°C.

### Enzyme-linked immunosorbent assay

Immulon 2HB (Thermo Scientific, #3455) plates were coated with 50 µL of 1 µg/mL of purified MACV GPC or JUNV GPC in PBS at 4°C overnight. The next day, ELISA plates were washed with PBS containing 0.1% Tween-20 (PBS-T) and blocked for an hour at room temperature on a plate shaker with 3% milk in PBS-T. Mouse serum was diluted in 1% milk in PBS-T, then serially diluted twofold. Plates were incubated with diluted mouse serum for 2 hours at room temperature. Plates were washed with PBS-T before adding a goat anti-mouse IgG (H + L) secondary antibody (HRP-conjugated) (Invitrogen, #31430), which was diluted in 1% PBS-T at 1:2,000. Plates were incubated at room temperature for 1 hour. SureBlue TMB 1 component substrate (KPL, #52-00-01) was then added to plates and quenched after 5 minutes with 250 mM HCl. Absorbance at 450 nm was immediately read on a SpectraMax 190 microplate reader (Molecular Devices, San Jose, CA).

Endpoint titer was determined from interpolated values based on an anti-His antibody standard (DSHB, N144/14) run on each plate. Samples lacking absorbance at our lowest dilution of 1:20 were assigned a titer of 1 to indicate a titer below the limit of detection. The mean endpoint titers for each sample were reported based on measurements from at least two technical replicates.

### Focus reduction neutralization test

To produce VSVΔG-RFP MACV or JUNV pseudotypes, HEK 293T cells were seeded at 5 × 10^6^ cells per 10 cm dish, incubated for 24 hours, and transfected using calcium phosphate with 25 μg of pCG1 MACV or pCG1 JUNV plasmid. After 24 hours of transfection, cells were infected for 2 hours with VSV-G pseudotyped VSVΔG-RFP at an MOI of ∼1–3. Pseudotypes were harvested by collecting the culture media 28–30 hours after infection. These supernatants were clarified by centrifugation twice at 6,000 × *g* and stored at −80°C.

For the neutralization assay, Vero E6 cells were seeded at a density of 2.5 × 10^4^ cells/well in 100 μL in a 96-well collagen-coated plate. Pseudotype virus (100–300 focus-forming units/well) was mixed with serum samples from a serial twofold dilution and incubated for 1 hour at 37°C. To neutralize any potential VSV-G carryover virus, mouse anti-VSV Indiana G antibody (1E9F9) was also added at a concentration of 600 ng/mL (Absolute Antibody, Ab01402-2.0), and the Vero E6 cell culture media was replaced with this serum-virus mixture. After 21–22 hours, Vero E6 cells were washed and fixed with 4% paraformaldehyde before visualization on an S6 FluoroSpot Analyzer (CTL, Shaker Heights, OH). Individual infected foci were enumerated, and the values were compared to control wells without antibody. The FRNT_50_ was measured as the greatest serum dilution at which focus count was reduced by at least 50% relative to control cells that were infected in the absence of serum. The geometric mean FRNT_50_ titers for each sample were reported based on readings from at least two technical replicates.

### Enzyme-linked immunospot assay

ELISpot plates (MilliporeSigma MultiScreen 96-Well Assay Plates for ELISpot Assays—MAIPS4510) were activated with 30% ethanol, then coated with Mouse IFN-γ capture antibody (BD Biosciences, #551881) at 4°C overnight. The next day, plates were washed and blocked with RPMI + 10% FBS for 2 hours at room temperature. Spleens were collected from immunized animals following euthanasia. Spleens were homogenized and passed through a 40-μm cell strainer (Falcon, 352340), followed by washes with 1× PBS and ACK lysis (Quality Biological, 118-156-101). Splenocytes from immunized mice were added to the plate at 300 k cells/well along with VSV N or MACV GPC peptides at 1 μg/mL and recombinant IL-2. The plate was incubated at 37°C, 5% CO_2_ for 20 hours. After incubation, the plate was washed with PBS-T (1× PBS + 0.05% Tween 20) and the biotinylated anti-mouse IFN-γ detection antibody (BD Biosciences, #551881) was added to wells for 2 hours. Plates were washed again, and then streptavidin-HRP (BD Biosciences, #557630) was added for 1 hour. Plates were then washed with PBS, and freshly prepared AEC substrate solution (BD Biosciences, #B551951) was added to the wells. Wells were monitored for 10–60 minutes for the appearance of spots, then the reaction was stopped by running the plate under tap water. After the plate dried, it was scanned with the S6 FluoroSpot Analyzer (CTL, Shaker Heights, OH). Individual spots were enumerated and compared to control wells without stimulus. The data were reported as the average of two technical replicates.

A peptide pool comprising 49 peptides spanning the entire MACV GP1 sequence was ordered from Genscript as 15 mers with 10 amino acid overlaps. In previous work (68), we evaluated separate peptide pools of 15-mers spanning either MACV GP1 (49 peptides) or GP2 (48 peptides). In preliminary experiments, we found that only the peptide pool from GP1 elicited a detectable T-cell response. Therefore, all subsequent experiments utilized only the GP1 peptides. The VSV N peptide used for stimulation is the immunodominant epitope RGYVYQGL.

### Statistical analyses

GraphPad Prism Version 10.6.1 was utilized for statistical analyses as detailed in the figure legends.

## Data Availability

All data are provided within the figures of the paper. Additional information is available from the corresponding author upon request.
